# A Pennsylvania Alumni Association of the American Veterinary College

**Published:** 1895-03

**Authors:** W. L. Rhoads

**Affiliations:** Secretary pro. tem.


### A PENNSYLVANIA ALUMNI ASSOCIATION OF THE AMERICAN VETERINARY COLLEGE.

A PRELIMINARY meeting of the graduates of the American Veterinary Colleges in Pennsylvania, under a call from State Secretary Rhoads, was held at 3452 Ludlow street, Philadelphia, on Friday evening, February 22, 1895, for the purpose of considering the necessary steps toward forming a State Alumni Association of the A. V. C.

A number of letters were read from graduates in various parts of the State, all favoring the movement and promising their support. After a general discussion of the movement temporary officers were elected as follows: Chairman, Dr. F. S. Allen; Secretary, W. L. Rhoads.

It was then decided that a meeting of the graduates be held not later than April, and that a moderate spread be prepared, and to elect permanent officers.

On motion, Drs. Hoskins, Allen and Bieber were appointed a Committee of Arrangements and instructed to prepare a suitable toast list for the occasion.

Also, that the permanent officers shall consist of President, Vice-president and Secretary-treasurer, the President having the power to appoint an Executive Committee.

W. L. Rhoads,
Secretary *pro. tem.*

